# Who is going to walk? A review of the factors influencing walking recovery after spinal cord injury

**DOI:** 10.3389/fnhum.2014.00141

**Published:** 2014-03-13

**Authors:** Giorgio Scivoletto, Federica Tamburella, Letizia Laurenza, Monica Torre, Marco Molinari

**Affiliations:** ^1^Spinal Cord Unit, IRCCS Fondazione S. LuciaRome, Italy; ^2^Clinical and Research Movement Analysis Lab, Fondazione S. LuciaRome, Italy

**Keywords:** spinal cord injury, walking recovery, prognostic factors

## Abstract

The recovery of walking function is considered of extreme relevance both by patients and physicians. Consequently, in the recent years, recovery of locomotion become a major objective of new pharmacological and rehabilitative interventions. In the last decade, several pharmacological treatment and rehabilitative approaches have been initiated to enhance locomotion capacity of SCI patients. Basic science advances in regeneration of the central nervous system hold promise of further neurological and functional recovery to be studied in clinical trials. Therefore, a precise knowledge of the natural course of walking recovery after SCI and of the factors affecting the prognosis for recovery has become mandatory. In the present work we reviewed the prognostic factors for walking recovery, with particular attention paid to the clinical ones (neurological examination at admission, age, etiology gender, time course of recovery). The prognostic value of some instrumental examinations has also been reviewed. Based on these factors we suggest that a reliable prognosis for walking recovery is possible. Instrumental examinations, in particular evoked potentials could be useful to improve the prognosis.

## Introduction

Walking recovery is one of the main goals of patients after SCI: walking is rated at first place (together with bladder and bowel function) at least by patients with incomplete lesions (Ditunno et al., 2008a). Furthermore, an epidemiological study shows an increase of the number of patients with incomplete lesions (e.g., with chances of walking recovery) (Pagliacci et al., 2003). Therefore, the recovery of ambulation has become the target of several pharmacological and rehabilitative approaches (Wernig and Muller, 1992; Domingo et al., 2012) and a precise evaluation of the natural recovery of walking and of the prognostic factors influencing this function has become mandatory (Steeves et al., 2007).

In the present work we reviewed the effect of several clinical and demographic features on the prognosis for walking recovery. Furthermore, because one of the main problems of the acute phase of SCI is the lack of reliable examinations, we considered the prognostic value of neurophysiological and neuroimaging examinations.

Finally, the effect of early pharmacological and surgical interventions on walking recovery will be examined.

## Materials and methods

A systematic search was performed of all papers as well as websites mentioning spinal cord injury and walking The literature search was conducted without time limits to identify papers that explicitly mentioned the walking capacity in patients with SCI. Databases included PubMed, Ovid MEDLINE, CINAHL, PsychINFO, Cochrane Central Register of Controlled Trials and Scopus, which includes Embase citations. All study designs, including case reports, were included, with no restrictions on the ages of participants. Non-English articles and animal studies were excluded. The following search terms were used: prognosis prediction, SCI, paraplegia/tetraplegia/quadriplegia, ambulation/gait and walking/walking capacity. In addition, other databases, such as Google and a hand search of Spinal Cord yielded other citations not identified by the above strategy.

Two authors (Giorgio Scivoletto and Federica Tamburella) independently identified and classified the papers through a review of the abstracts, texts, and references and circulated them to the authors' panel.

## Clinical examination

**Figure 1:**
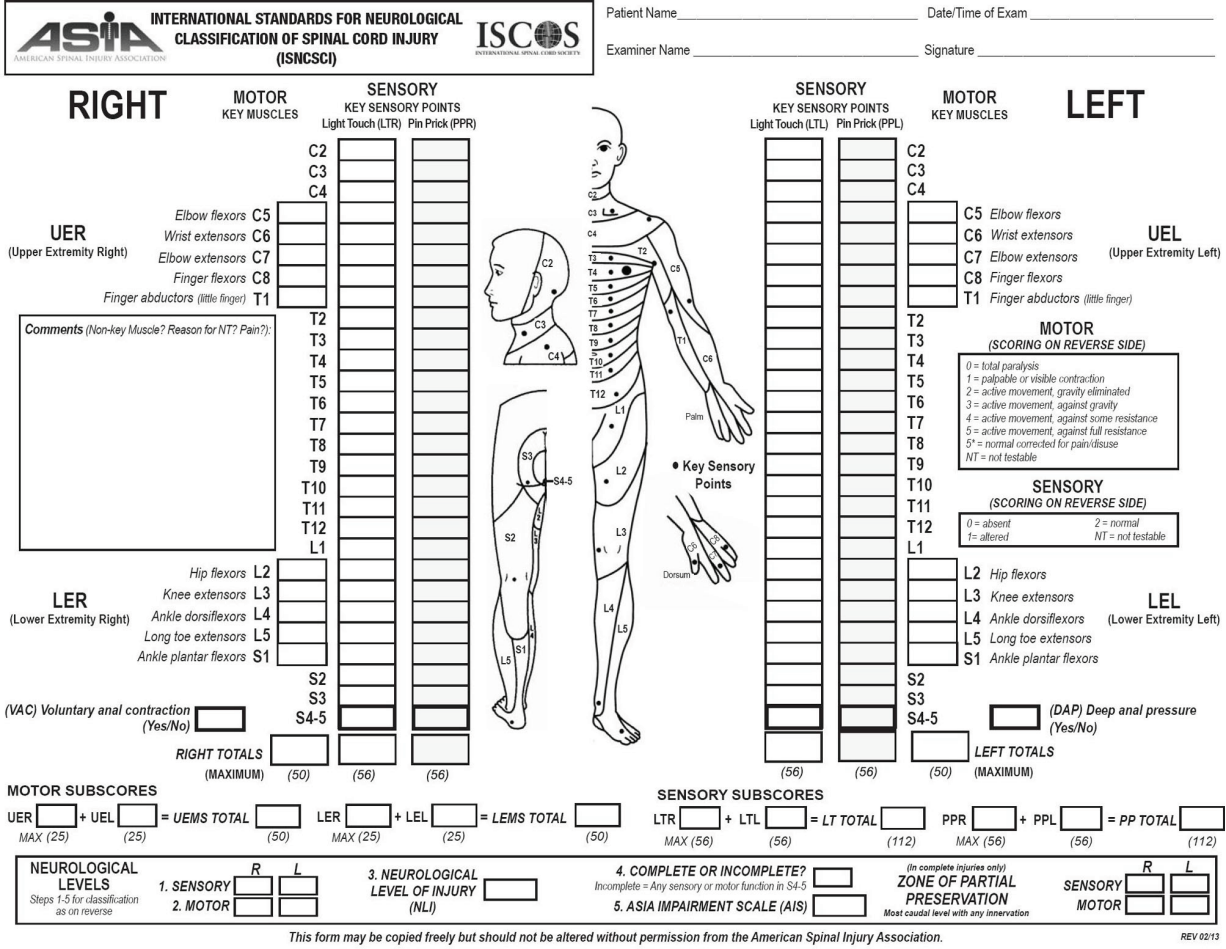
**Scoring sheet for the International Standards for Neurological Classification of Spinal Cord Injury**. American Spinal Injury Association: International Standards for Neurological Classification of Spinal Cord Injury, revised 2013; Atlanta, GA. Reprinted 2013.

**Figure 2:**
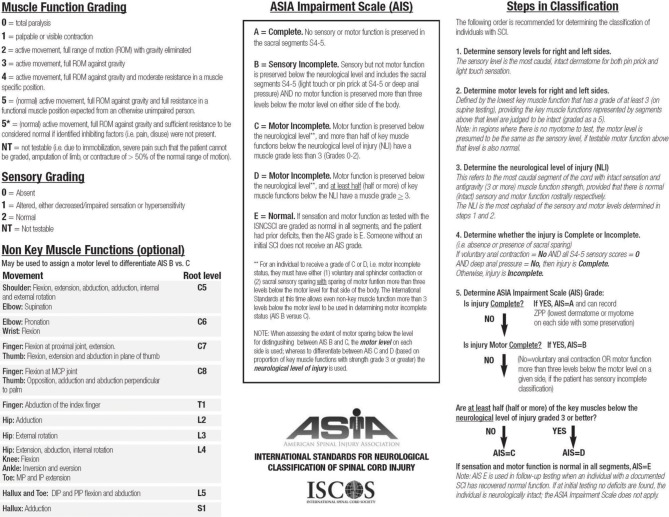
**Scoring sheet for the International Standards for Neurological Classification of Spinal Cord Injury**. American Spinal Injury Association: International Standards for Neurological Classification of Spinal Cord Injury, revised 2013; Atlanta, GA. Reprinted 2013.

The most relevant prognostic factor for functional recovery in SCI patients is the neurological status at the moment of the first examination. The physical examination of these patients has been standardized by the American Spinal Injury Association in the International Standards for Neurological Classification of Spinal Cord Injury (ISNCSCI) (American Spinal Injury Association, 2000). Based on this examination it is possible to establish the neurological level of injury, as well as the severity of the lesion (impairment). Components also include a rectal examination for voluntary anal contraction and anal sensation (Figures 1, 2). Patients are considered to have a complete lesion (AIS impairment A), according to the ASIA Impairment Scale (AIS), in the absence of sensory or motor function at the lowest sacral segments. Incomplete lesions are defined when sensation and/or motor function are preserved below the neurologic level of injury, and in particular in the lowest sacral segments (anal sensation, including deep anal pressure and voluntary external anal sphincter contraction) (Figure 2).

This examination should usually be performed at 72 h after the lesion because this timing seems to have a more accurate prognostic value than earlier assessment (Herbison et al., 1991).

## AIS grade conversion and walking recovery

For the aim of this review we would define walking recovery as the regained ability to walk independently in the community, with or without the use of devices and braces. This is also defined “functional walking” and has been described by several authors (Hussey and Stauffer, 1973) as the capacity to walk reasonable distances both in and out of home unassisted by another person.

For a long time AIS grade conversion has been considered the basis to predict the possibility of achieving functional walking. However, a recent article by van Middendorp et al. (2009) questioned the relationship between AIS grade conversion and ability to walk as we will show below.

Patients with AIS impairment A (motor and sensory complete lesion) at their first examination have very few chances of neurological recovery below the lesion. When the examination is performed at 72 h post-injury, 80% of the initial AIS A patients remain as AIS A, with about 10% converting to AIS B (i.e., some sensory function) and about 10% converting to AIS C (with some motor recovery below the lesion) (Burns et al., 2012). However, if the first examination is performed later, the percentage of improvement decreases dramatically to 2.5% (Scivoletto et al., 2004a) (Table 1). Accordingly, the possibility of patients with AIS impairment A of achieving functional walking is very limited too. Furthermore, also between the patients who converted to an incomplete lesion only 14% recovered some walking function (van Middendorp et al., 2009). The AIS A patients who achieve some walking function usually are low thoracic or lumbar levels (T12-L3) and need braces and devices to walk (Ditunno et al., 2008b; Table 2). Finally, these patients are usually limited ambulators, with slow average velocities and great energy expenditure (Vaccaro et al., 1997).

**Table 1:** **Prediction of recovery according to AIS impairment scale**.

**AIS grade at admission**	**A**	**B**	**C**	**D**
**First examination at 72 h^10^**	**One-year follow-up AIS grade**			
A	84%	8%	5%	3%
B	10%	30%	29%	31%
C	2%	2%	25%	67%
D	2%	1%	2%	85%
**First examination at 30 days^11^**	**One-year follow-up AIS grade**			
A	95%	0	2,5%	2,5%
B	0	53%	21%	26%
C	1%	0	45%	54%
D	2%	0	0	96%

**Table 2:** **Prediction of functional walking according to AIS impairment and other features**.

**AIS/lesion level at admission**	**Functional walking/authors (references)**
AIS A/cervical lesion	0% (Waters et al., 1994a,b)
	0% (Ditunno et al., 2008b)
AIS A/thoracic and lumbar lesions	5% (Waters et al., 1994a,b)
	8.5% (Ditunno et al., 2008b)
**AIS at admission and sensation**	**% recovery of community ambulation at 1 year post-injury/authors (references)**
AIS B (only light touch preservation)	0% (Waters et al., 1994a,b)
	11% (Crozier et al., 1991)
	33% (Waters et al., 1994a,b)
AIS B (light touch + pin prick preservation)	89% (Crozier et al., 1991)
	66% (Foo et al., 1981)
	75% (Katoh and el Masry, 1995)
**AIS at admission and age**	**% recovery of community ambulation at 1 year post-injury/authors (references)**
AIS C < 50 years	91% (Burns et al., 1997)
	71% (Scivoletto et al., 2003)
AIS C > 50 years	42% (Burns et al., 1997)
	25% (Scivoletto et al., 2003)
AIS D < 50 years	100% (Burns et al., 1997)
	100% (Scivoletto et al., 2003)
AIS D > 50 years	100% (Burns et al., 1997)
	80% (Scivoletto et al., 2003)

AIS grade B patients (those with motor complete, sensory incomplete lesion at 72 h examination) usually show some motor recovery and they can convert to AIS C or even AIS D grade. However, the overall recovery of ambulation is considered to be about 33% (Katoh and el Masry, 1995; van Middendorp et al., 2009). The percentage of walking recovery may vary depending on the modality of the sensation spared at the lowest sacral segments. Several studies reported a relationship between pinprick preservation and recovery in AIS B patients. AIS grade B patients with pinprick preservation have a better walking recovery than those with light touch only (Foo et al., 1981; Crozier et al., 1991; Waters et al., 1994a; Katoh and el Masry, 1995; Oleson et al., 2005) (Table 2). This finding has an anatomical basis at the spinal cord level. The preservation of pinprick perception together with light touch one indicates less extensive damage to the spino-thalamic tracts and posterior column. Therefore, in these cases, there is a high likelihood of some sparing of the motor pathways conveyed by the nearby cortico-spinal tracts (Oleson et al., 2005).

Motor incomplete (AIS C) patients have a better prognosis for walking recovery than sensory incomplete ones. The overall rate of recovery is about 75% (Maynard et al., 1979; Crozier et al., 1992; Waters et al., 1994b; van Middendorp et al., 2009). This percentage includes both the patients who converted to AIS D and those who remained AIS C but achieve at least some walking function (van Middendorp et al., 2009); these patients probably have low thoracic or lumbar lesions and walk with braces and devices. Several factors may influence the chance of walking recovery in these patients: lower extremity strength, motor recovery timing, age and upper extremity strength for tetraplegic patients are the most important ones (Crozier et al., 1992; Waters et al., 1994b). In AIS C patients age seems to be a strong prognostic factor for walking recovery. Age represents a clear negative prognostic factor for walking recovery: AIS C subjects younger than 50 years have a chance of achieving functional walking of 80–90%, but this percentage dramatically decreases to 30–40% in older patients (Table 2) (Perot and Vera, 1982; Foo, 1986; Burns et al., 1997; Scivoletto et al., 2003). Different hypotheses have been offered to explain the negative effect of age. The functional potential for a given neurological deficit is lower at older age; this may be considered reasonable since functional abilities generally decline as people's age increases. In normal ageing “reserve (peak) capacity” (or “vitality”) (DiGiovanna, 2000) seems to peak at around 30 years of age, and then gradually declines until death. Disease processes, including SCI and its complications, are considered to accelerate this process of decline. Jakob et al. (2009) offers another possible explanation. In his study he found that age is not correlated with neurologic recovery, but is correlated with a worse functional outcome in terms of independence in daily life activities and walking function. He therefore suggested that the neurological recovery is not directly related to the functional outcome and that elderly patients have difficulties in translating neurological recovery into positive functional changes.

Finally, AIS D patients at admission have very good ambulation prognosis at 1 year post-injury (Burns et al., 1997; Scivoletto et al., 2003). All patients, regardless of age, who initially were classified as AIS D (within 72 h) were able to walk at the time of discharge from inpatient rehabilitation (Burns et al., 1997; van Middendorp et al., 2009).

## Other clinical factors

In addition to AIS grade, several other factors evaluated at 72 h after the lesion have been considered in the prognosis of walking recovery and are examined below.

### Reflexes

In the very early examination of SCI patients the presence/absence of the delayed plantar response (DPR) must be assessed. DPR is characterized by a delayed response to an unusually strong stimulus to the sole of the foot (Weinstein et al., 1997). The onset of this response following the stimulus could be 500 ms or a full second following the initiation of the stimulus (Weinstein et al., 1997). The DPR shows a reciprocal relationship with the Babinski sign and it is particularly relevant because it allows the prognosis during the spinal shock phase (Ko et al., 1999). The DPR is a negative prognostic indicator as it is more often present and lasts longer (more than 1 day) in SCI patients who do not recover any voluntary movement (Weinstein et al., 1997; Ko et al., 1999).

### Syndromes

Based on the distribution of sensory and motor loss, the ISNCSCI allow to identify several incomplete spinal cord syndromes with different prognostic values.

The *central cord syndrome (CCS)* is mostly seen following cervical lesion. It represents about 9% of the total SCIs and 44% of the clinical syndromes (McKinley et al., 2007) and is characterized by a greater involvement of the upper extremities than the lower extremities. The CCS is a clinical picture that recognizes several causes (with and without bone injury) and several different mechanisms (including direct injury of the spinal cord or vascular injuries) (McKinley et al., 2007) that primarily affects the center of the spinal cord and generally has a favorable prognosis as to independence in daily life activities and bladder and bowel function recovery (Newey et al., 2000; Dvorak et al., 2005; Aito et al., 2006). Because of the lesser involvement of the lower extremities, CCS is considered to have a good prognosis for walking recovery too (Merriam et al., 1986; Penrod et al., 1990; Roth et al., 1990; Burns et al., 1997; Aito et al., 2006). The percentage of patients who recover walking varies from 40 to 97%, but is strongly influenced by age. Several studies confirm that younger patients (less than 50 years old) have twice the chance of achieving independent walking than older ones (Foo, 1986; Merriam et al., 1986; Penrod et al., 1990; Roth et al., 1990; Burns et al., 1997; Newey et al., 2000; Dvorak et al., 2005; Aito et al., 2006).

The *Brown-Séquard syndrome (BSS)* is characterized by ipsilateral hemiplegia and contralateral hemianalgesia due to spinal hemisection (Brown-Sequard, 1868). It accounts for 2–4% of all traumatic SCIs and 17% of the clinical syndromes (McKinley et al., 2007). The pure form of BSS is rarely seen and the Brown-Séquard Plus Syndrome (relative ipsilateral hemiplegia with a relative contralateral hemianalgesia) is much more frequent (Roth et al., 1991). BSS is more frequent at cervical level and is usually associated with stab-wound injuries (Gentleman and Harrington, 1984). BSS is characterized by a good functional prognosis. About 75% of patients achieve independent walking at discharge from rehabilitation (Stahlman and Hanley, 1992). In this framework an important predictor for walking recovery is the distribution of the impairment: if the upper limb is weaker than the lower limb, then patients are more likely to ambulate at discharge (Kirshblum and O'Connor, 1998).

The *anterior cord syndrome* is due to a lesion that involves the anterior two thirds of the spinal cord and preserves the posterior columns (Maynard et al., 1997), and account for 1% of all the SCIs and 5% of the clinical syndromes (McKinley et al., 2007). It may derive from a retropulsed disc or bone fragments (Bauer and Errico, 1991), direct injury to the anterior spinal cord, or with lesions of the anterior spinal artery that provides the blood supply to that tract of spinal cord (Cheshire et al., 1996). Lesions of the anterior spinal artery may result from diseases of the aorta, cardiac or aortic surgery, embolism, polyarteritis nodosa, or angioplasty (Cheshire et al., 1996). Anterior cord syndrome is characterized by a variable loss of motor as well as pinprick sensation with a relative preservation of light touch, proprioception, and deep-pressure sensation. Due to the massive involvement of the anterior and lateral spinal cord with inclusion of the cortico-spinal tracts, only 10–20% of the patients with an anterior cord syndrome have the chance to recover muscle function, and even in those with some recovery, usually motor strength is low and coordination is lacking; consequently these patients have low walking recovery chances (Bohlman, 1979).

#### Etiology of the lesion

Most of the literature on SCI is focused on the rehabilitation of traumatic patients, despite the relevant incidence of non-traumatic lesions, considered to account for a percentage of the total SCIs varying from 30 to 80% (Buchan et al., 1972; Celani et al., 2001; Citterio et al., 2004). Patients with non-traumatic lesions differ from their traumatic counterparts for several prognostic factors. They are usually older, with a more even distribution of genders and a higher frequency of incomplete lesions. Therefore, a direct comparison of these two populations is difficult (Scivoletto et al., 2011). However, when the confounding effect of these factors is eliminated by means of statistics, patients with non-traumatic spinal cord lesions can achieve comparable rates of functional gains as their traumatic spinal cord injury counterparts (McKinley et al., 2000, 2001; Mckinley et al., 2002). With regard to walking function, recently a number of articles compared the recovery of ambulation in traumatic and non-traumatic SCIs and found that the two populations achieve comparable walking capacity with an overall percentage of patients varying from 35 (Scivoletto et al., 2011) to 49% (Marinho et al., 2012).

### Gender

There are only few studies on gender related differences in neurological and functional outcomes after inpatient rehabilitation of SCI (Greenwald et al., 2001; Scivoletto et al., 2004b; Sipski et al., 2004). Two of them (Greenwald et al., 2001; Scivoletto et al., 2004b) found no significant differences between the two genders with regard to daily life independence, motor efficiency, American Spinal Injury Association motor scores (Greenwald et al., 2001) and walking function (Scivoletto et al., 2004b). However, Sipski et al. (2004) found gender-related differences in daily life independence, but did not specifically focus on walking recovery. Women with SCI may have more natural neurologic recovery than men, but, for a given level and degree of neurologic injury, men tend to do better functionally than women at time of discharge from rehabilitation (Sipski et al., 2004).

## Formulas and algorithms

In the last three decades several attempts have been made to link one or more of the above mentioned factors (and of the results of instrumental examinations discussed below) to the prognosis for walking recovery.

Waters et al. (1994b) examined the relationship between lower extremity strength at first examination in incomplete paraplegics and walking recovery: all patients with an initial (1-month) lower extremity motor score of ≥10 points ambulated in 1 year. Seventy percent of patients with an initial motor score between 1 and 9 ambulated at 1 year. Furthermore, all patients with an initial hip flexor or knee extensor Grade ≥2 ambulated in the community at 1 year.

The same author examined the odds of walking recovery in incomplete tetraplegics and found that, although the relationship between initial lower extremity motor score and walking holds true for tetraplegics, these patients have less chance to achieve ambulation (Waters et al., 1994a): 63% of the patients with an initial lower extremity motor score of ≥10 points ambulated by 1 year, vs. 21% of those with an initial motor score between 1 and 9 (Waters et al., 1994a). In addition, Waters stressed the relationship between upper extremities strength and ambulation recovery in tetraplegics: patients who are community or household ambulators have significant higher motor scores. The author linked this datum to the importance of upper extremities strength for devices use during walking (Waters et al., 1994a).

Crozier et al. (1992) focused on the timing of recovery of lower extremity motor strength and concluded that early recovery of quadriceps strength is an excellent prognostic factor for ambulation. All patients with an initial quadriceps strength of at least Grade 2/5 who attained a grade of ≥3/5 in at least one quadriceps by 2 months post-injury achieved functional ambulation (ability to walk independently in the community, with or without the use of devices and braces) at follow-up. However, only 25% of those who did not recover quadriceps strength of 3/5 within 2 months were able to walk at follow-up.

More recently, Zörner et al. (2010) developed an algorithm based on outcome predictors and aimed at identifying subgroups of patients in the sub-acute phase who could achieve functional walking. For patients with incomplete paraplegia, lower extremity motor scores, pinprick scores and age were the best predictors for walking recovery. For patients with incomplete tetraplegia the more reliable predictors were the lower extremity motor scores, the tibial SSEP score and the AIS grade.

In 2011 van Middendorp et al. (2011) produced a simple clinical prediction rule based on the combination of age (<65 vs. ≥65 years), motor scores of the quadriceps femoris (L3), gastrocsoleus (S1) muscles, and light touch sensation of dermatomes L3 and S1. This rule showed an excellent discrimination capacity in recognizing patients who achieved independent ambulation (ability to walk independently, with or without braces and orthoses for <10 m) at follow-up from those who were dependent walkers or non-walkers.

## Instrumental examination

### Somatosensory evoked potentials (SSEPs) (Table 3)

SSEPs are used for clinical diagnosis in patients with neurologic disease, and many studies have been performed to determine the value of SSEPs in the prediction of walking recovery in SCI patients (Young and Dexter, 1979; Kaplan and Rosen, 1981; Young, 1985; Foo, 1986; Ziganow, 1986; Katz et al., 1991; Aalfs et al., 1993; Jacobs et al., 1995; Curt and Dietz, 1997).

**Table 3:** **Prognostic value of SSEPs and MEPs**.

	**Six months walking capacity**			
	**Normal (%)**	**Functional (%)**	**Therapeutic (%)**	**No walking (%)**
**LOWER LIMBS SSEPS AND AMBULATION** (Curt and Dietz, 1997)				
**Intial SSEP evaluation**				
Normal	83	17	0	0
Present, altered	10	60	10	20
Absent	0	7	13	80
**LOWER LIMBS MEP AND AMBULATION** (Curt et al., 1998)				
**Intial MEP evaluation**				
Normal	100	0	0	0
Absent	11	0	78	

Most of these studies conclude that early SSEPs can predict motor improvement and ambulation outcome in SCI patients. However, SSEPs do not seem to offer additional prognostic accuracy if compared to clinical examination according to the ISNCSCI for both complete and incomplete patients (Young and Dexter, 1979; Kaplan and Rosen, 1981; Perot and Vera, 1982; Chabot et al., 1985; Katz et al., 1991; Aalfs et al., 1993; Curt and Dietz, 1997).

When a reliable clinical examination, together with the ISNCSCI is impossible (patients unresponsive, for example because sedated or under the effect of alcohol or drugs, or uncooperative, for example because of pain) then SSEPs are helpful to determine if they have SCI (Curt and Dietz, 1997). In addition, SSEPs may be helpful to differentiate between SCI and hysteric paraplegia, a differential diagnosis that may be very difficult (Kaplan et al., 1985).

#### Motor evoked potentials (MEPs) (Table 3)

Transcranial magnetic stimulation allows an examination of the conductivity of the motor tracts following cortical or spinal lesions in humans. According to a study of Curt, MEPs can contribute toward diagnosing lesions of different neurologic structures within the spinal cord and in predicting the recovery of functional movements (Curt et al., 1998). The study shows that MEPs recordings are sensitive to indicate motor tract lesions in approximately 90% of SCI patients and predictive for the recovery of upper and lower limb motor function. In this sense they are of similar prognostic value to clinical examination in the prediction of functional recovery. MEPs can be used in combination with the ASIA protocol to follow the recovery of clinical motor functions in relation to that of descending motor tracts for impulse transmission. In Curt's study, MEPs were highly predictive of ambulatory capacity. All patients with elicitable MEPs at initial examination recovered a muscle strength of 3/5 or more of the respective muscles. Not surprisingly, MEPs recordings in SCI patients are more sensitive than SSEPs recordings for revealing the involvement of motor tract fibers and are at least as sensitive as the ASIA protocol in predicting the resulting functional deficit. Similarly to SSEPs, the use of MEP recordings is mostly appropriate in patients who are uncooperative (approximately 15% of patients with acute SCI) (Bozzo et al., 2011).

#### Magnetic resonance imaging (Table 4)

Before the advent of MRI, there were no imaging methods to assess the severity of traumatic SCI. MRI provides a rapid non-invasive means of evaluating the condition of spinal cord parenchyma and depicting the injured spinal cord and accurately showing the extent of macroscopic damage (Yamashita et al., 1991). It should be noted, however, that to the best of our knowledge, no study examined the relationship between MRI aspect and walking recovery, but only with neurologic recovery (AIS grade conversion) that is only partially related to walking (see above).

**Table 4:** **MRI and lesion severity**.

**Authors**	**Results**
**PRESENCE OF HEMORRHAGE AT INITIAL EXAMINATION**	
Marciello et al., 1993	Hemorrage = low upper extremity and no lower extremity recovery
Flanders et al., 1990	Hemorrage = decreased motor power, lower motor recovery rate, and fewer muscles with useful function
Ramón et al., 1997	Hemorrage = complete injury
**SIZE OF HEMORRHAGE**	
Boldin et al., 2006;	
Flanders et al., 1990;	Small hemorrhage = higher recovery rates
Schaefer et al., 1992	
Bondurant et al., 1990;	No relationship between hemorrhage size and recovery
Flanders et al., 1996	
**PRESENCE OF EDEMA**	
Flanders et al., 1996	Edema = prognosis of recovery to functional levels (D/E)
Ramón et al., 1997	Edema = association with incomplete syndromes
**SIZE OF EDEMA**	
Flanders et al., 1990;	Degree of edema is inversely proportional to initial impairment and future recovery
Flanders et al., 1996;	
Ramón et al., 1997	
Boldin et al., 2006; Flanders et al., 1990	Multiple levels involvement = poorer prognosis and greater chance of complete lesions
Flanders et al., 1996	Involvement of only one to three segments = improved prognosis

For prognostic purposes the T2 sagittal images seem to be the most useful ones, while T1 and axial images do not correlate with the prognosis (Bozzo et al., 2011). A damaged spinal cord exhibits a variable amount of intramedullary hemorrhage and edema. Both the presence of these two features and the amount of parenchyma that is affected by hemorrhage and edema are directly related to the degree of initial neurologic deficit and to the prognosis (Bondurant et al., 1990; Flanders et al., 1990). Based on these aspects, Bondurant and associates (Bondurant et al., 1990) proposed a classification which consider four different MRI patterns: Pattern 1 shows a normal MRI signal in the cord; pattern 2 represents single-level edema; pattern 3 is multi-level edema; and pattern 4 is mixed hemorrhage and edema.

Most studies showed that patients with spinal cord hemorrhage will have decreased motor power, lower motor recovery rates, and fewer muscles with useful function, 1 year after injury in comparison with subjects with small, non-hemorrhagic lesions (Bondurant et al., 1990; Flanders et al., 1990, 1996; Yamashita et al., 1991; Schaefer et al., 1992; Marciello et al., 1993; Sato et al., 1994; Ramón et al., 1997); hemorrhage on initial MRI (within 15 days from the lesion) is associated with a complete injury in almost 100% of the patients (Ramón et al., 1997). If no hemorrhage is seen on initial MRI, patients will have an incomplete lesion and have a significantly better prognosis for motor recovery in the upper and lower extremities, as well as improvement in their Frankel and/or ASIA impairment scale classification (Schaefer et al., 1992).

It is unclear whether the size of the hemorrhage is a prognostic feature. Some authors (Flanders et al., 1990; Schaefer et al., 1992; Boldin et al., 2006) have shown that small hemorrhages may offer higher recovery rates; others showed no difference based on the size of the hemorrhage (Bondurant et al., 1990; Flanders et al., 1996).

With regard to spinal cord edema, this MRI finding seems to have a good prognostic value. In incomplete SCIs, the finding of edema in MRI is associated with a good prognosis of neurological recovery (Flanders et al., 1996). Furthermore, the incomplete syndromes, such as the Brown-Sèquard syndrome, seem to be associated with the edema pattern (Ramón et al., 1997). However, if the edema involves multiple levels, it tends to be associated with a poorer prognosis and a greater chance of having a complete lesion (Flanders et al., 1996; Boldin et al., 2006). If the cord edema is limited to one to three segments only, then the lesion is usually milder in nature, with an improved prognosis (Bauer and Errico, 1991).

Based on the classification of Bondurant et al. (1990), Bozzo et al. (2011) reviewed the data of several articles (Schaefer et al., 1992; Shimada and Tokioka, 1999; Andreoli et al., 2005) and found a correlation with the AIS conversion of patients. As already reported hemorrhage is the more severe MRI aspect, with about 95% of patients remaining with the same AIS grade of admission examination. Patients with diffuse edema also showed a poor improvement, as only 28% of them showed an improvement of AIS grade. Conversely, patients with single level edema pattern showed a good neurological outcome as 90% of them improved for a mean of 1.9 AIS grades.

Other positive correlations have been described: greater degree of cord compression, greater degree of canal compromise, and the severity of soft tissue injuries seem to be all associated with poorer neurological outcomes (Flanders et al., 1996; Selden et al., 1999; Dai and Jia, 2000; Miyanji et al., 2007; Song et al., 2008).

## Treatment

In the last decade several interventions aiming at reducing the spinal cord damage (neuroprotection) have been proposed (Becker and McDonald, 2012). However, these interventions are still at an experimental level (Becker and McDonald, 2012). Therefore, in the following paragraphs we will focus only on the use and efficacy of high dose methylprednisolone (which, although questioned, is still the most widely used pharmacological treatment in the acute phase of SCI) and of early surgical intervention. It should be noticed that in both cases, studies referred to neurological improvement rather than to walking recovery. Therefore, data on the efficacy of these treatments on ambulation are not available.

### Methylprednisolone

The administration of high-dose methylprednisolone (MP) to patients with spinal cord injuries has been reported in the National Acute Spinal Cord Injury Studies (NASCIS, NASCIS-II, and NASCIS-III) (Bracken et al., 1984, 1990, 1997). Since then, the use of MP increased and became a standard of care for acute traumatic SCIs (Hurlbert, 2001). It has been hypothesized that MP attenuates the inflammatory cascade and lessens lipid peroxidation, thus decreasing secondary Spinal Cord damage (Delamarter et al., 1995). In the NASCIS studies, the 24 and 48 h administration of high dose MP produced an important neurologic recovery (AIS grade improvement) paralleled by a functional amelioration (Bracken et al., 1997). However, several recent revisions of NASCIS protocols and other randomized trials questioned the efficacy of steroids administration to achieve a neurologic improvement (Hurlbert, 2001; Matsumoto et al., 2001; Suberviola et al., 2008; Bydon et al., 2013). Furthermore, the 48-h–infusion of MP seems to be associated with an increased risk of pneumonia, sepsis, gastrointestinal bleeding, and steroid myopathy (Pointillart et al., 2000; Quian et al., 2004).

Based on these evidences, both the Consortium for Spinal Cord Medicine clinical practice guidelines (Consortium for Spinal Cord Medicine, 2007) and the neurosurgical guidelines (2002) consider the use of high-dose MP to be a treatment option rather than a standard.

### Surgery trials

The undisputed benefits of surgical treatment for unstable vertebral injuries include decreased hospital stay, fewer sequelae from prolonged immobilization, and more rapid admission to the rehabilitation system (Raineteau and Schwab, 2001).

Despite these evidence, the timing of decompression of the neural elements, and, in particular, the efficacy of early decompression (within 24 h) in improving neurologic recovery is still a matter of debate (Fehlings and Tator, 1999; Fehlings and Perrin, 2005). A meta-analysis of studies of early decompression from 1966 through 2000 (La Rosa et al., 2004), showed that surgery performed within 24 h produced a significant improvement in neurological recovery compared with late surgery, but concluded that the evidence was not strong and that early surgery could be considered only as a practice option.

Starting from this framework, a recent prospective multicentric study (Fehlings et al., 2012) demonstrated that the odds of achieving a 2 AIS grade improvement is 2.8 times higher in patients undergoing early surgical decompression (within 24 h). However, a recent meta-analysis (van Middendorp et al., 2013) reported a lack of statistical robustness of the articles examined, therefore the relationship between early surgery and better neurological outcome is still to be demonstrated.

## Discussion

This review demonstrates that the chance of walking recovery after a SCI can be accurately predicted on the base of demographic data and clinical examination. Patients with complete sensory-motor lesions have very limited possibility of achieving walking function at follow up, and also if they are able to ambulate they usually are “limited ambulators.” The chances of walking recovery improve in less severe lesions, as demonstrated by AIS B and C subjects. AIS B patients can recover walking especially if their clinical picture shows a less severe involvement of the spinal cord (light touch and pinprick conservation = some sparing of the spino-thalamic and posterior columns tracts = higher possibility of cortico-spinal tracts preservation). Finally, subjects with AIS C lesions are bound to walk, especially the younger ones. This prognosis for walking may be sustained and empowered by instrumental examinations that help to assess the severity of the lesion and, in some cases (SSEPs and MEPs) are directly correlated with walking function.

The need to predict outcome based on expected neurological recovery and associated functional recovery has been emphasized as essential for health care planning (Ditunno, 1999) and this need is partially unmet.

During the first few days after SCI, definitive management strategies are formulated, which often include aggressive surgical decompression of the spinal cord (Wilson et al., 2012). This is also the time of greatest anguish for an injured patient and their family as they face significant prognostic uncertainty. A precise knowledge of the prognosis makes it possible to answer questions regarding function that patients usually ask after spinal cord injury: “Will I walk again?” and “What will I be able to do?” Furthermore, in countries with health care systems based on insurance, rehabilitation professionals have to justify and fight for appropriate services; furthermore they have to know how to allocate resources. Therefore, predicting recovery has become a rehabilitative imperative (Ditunno, 1999).

Finally, better knowledge of the course and prognosis of recovery after SCI and an understanding of the underlying mechanisms would help in the development of strategies and treatments to enhance neurological recovery. The number of interventions, therapies, and devices that have been developed and proposed to improve functional outcomes after SCI is enormous; several of these proposal will undergo clinical trials in the near future. Some early stage SCI clinical trials have recently been started and some experimental therapies have been introduced into clinical practice without a clinical trial being completed. Prognostic data are essential to evaluate the efficacy of new drugs and therapies (for example to distinguish between the natural recovery and the effect of treatments) and to project the clinical trials (for example to calculate the number of patients needed to obtain statistical power) (Fawcett et al., 2006).

## Limitations

This article has several limitations due to the nature of the works examined. Some of them are based on small sample sizes and the definition of walking function and of follow up time points vary across the studies. Furthermore, these articles mainly represent the experience from USA and, in part, Europe. Therefore, they do not reflect the whole world standards of care. As SCI management may differ in different geographical areas, the rates of recovery of walking could vary to. Finally, the distribution in time of the works examined is not regular. Although the study of the prognostic factors is still a matter of interest, most of the articles related to clinical factors date back to the 80 s and 90 s. Some prognostic factors may change over time as SCI management evolves. Based on these limitations the results of these studies could not be necessarily generalizable However, the factors that we examined here are still considered the base of the prognosis of SCI outcome (Burns et al., 2012).

## Funding

Supported in part by grant RC12G of the Italian Ministry of Health and grant P133 of the International Foundation for Research in Paraplegia to Giorgio Scivoletto.

### Conflict of interest statement

The authors declare that the research was conducted in the absence of any commercial or financial relationships that could be construed as a potential conflict of interest.
